# Principles and implementation strategies for equitable and representative academic partnerships in global health informatics research

**DOI:** 10.1093/jamia/ocaf015

**Published:** 2025-02-13

**Authors:** Elizabeth Campbell, Oliver J Bear Don’t Walk, Hamish Fraser, Judy Gichoya, Kavishwar B Wagholikar, Andrew S Kanter, Felix Holl, Sansanee Craig

**Affiliations:** Department of Environmental Health and Engineering, Johns Hopkins Bloomberg School of Public Health, Baltimore, MD 21205, United States; Center for Outbreak Response Innovation, Johns Hopkins Bloomberg School of Public Health, Baltimore, MD 21202, United States; Department of Biomedical Informatics, Columbia University Irving Medical Center, New York, NY 10032, United States; Department of Biomedical Informatics and Medical Education, University of Washington, Seattle, WA 98195, United States; Brown Center for Biomedical Informatics, Brown University, Providence, RI 02912, United States; Department of Radiology and Imaging Sciences, Emory University School of Medicine, Atlanta, GA 30322, United States; Harvard Medical School, Boston, MA 02115, United States; Department of Biomedical Informatics, Columbia University Irving Medical Center, New York, NY 10032, United States; DigiHealth Institute, Neu-Ulm University of Applied Sciences, Neu-Ulm 89231, Germany; Leibniz Science Campus Digital Public Health, Bremen, Germany; Department of Biomedical and Health Informatics, Children’s Hospital of Philadelphia, Philadelphia, PA 19146, United States

**Keywords:** global health informatics, health equity, stakeholder engagement, scalability and sustainability, community participation

## Abstract

**Objective:**

Developing equitable, sustainable informatics solutions is key to scalability and long-term success for projects in the global health informatics (GHI) domain. This paper presents key strategies for incorporating principles of health equity in the GHI project lifecycle.

**Materials and Methods:**

The American Medical Informatics Association (AMIA) GHI Working Group organized a collaborative workshop at the 2023 AMIA Annual Symposium that included the presentation of five case studies of how principles of health equity have been incorporated into projects situated in low-and-middle-income countries and with Indigenous communities in the U.S. and best practices for operationalizing these principles into other informatics projects.

**Results:**

We present five principles: (1) Inclusion and Participation in Ethical, Sustainable Collaborations; (2) Engaging Community-Based Participatory Research Approaches; (3) Stakeholder Engagement; (4) Scalability and Sustainability; (5) Representation in Knowledge Creation, along with strategies that informatics researchers may use to incorporate these principles into their work.

**Discussion:**

Presented case studies and subsequent focus groups yielded key concepts and strategies to promote health equity that may be operationalized across GHI projects.

**Conclusion:**

Equitable, sustainable, and scalable GHI projects require intentional integration of community and stakeholder perspectives in project development, implementation, and knowledge creation processes.

## Introduction

Global health informatics (GHI) is a branch of biomedical informatics that focuses on utilizing e-Health practices, including EHRs, mHealth, and telehealth, to provide resources, services, and information to improve population and clinical health outcomes for patients in low-and middle-income countries (LMICs), as well as medically underserved patients in high-resource settings.^1–3^ The American Medical Informatics Association (AMIA) Global Health Informatics Working Group (GHI-WG) is a collaborative of informaticians that focuses on knowledge sharing and broad collaboration in applying informatics to improve health outcomes worldwide. The group facilitates the exchange of information and expertise, supports the global development and implementation of informatics projects, and promotes best practices, standards, and evidence generation. This working group includes professionals from various backgrounds, such as healthcare providers, researchers, software developers, educators, community leaders, and policymakers, all working together to leverage informatics for global health improvement. The following perspective piece presents findings from a workshop organized by the AMIA GHI-WG and held at the 2023 AMIA Annual Symposium. The workshop centered on incorporating principles of health equity into the GHI project pipeline and how these findings may be operationalized across diverse GHI settings, with a focus on academic GHI collaborations. In this paper, we define health equity as the absence of systemic disparities in the social determinants of health, access to healthcare, and resources between advantaged and disadvantaged groups so that the highest level of health may be achieved for all individuals.^4^ GHI interventions have significant potential to empower patients, improve healthcare access, and expand technology utilization for the most vulnerable communities worldwide. However, numerous obstacles exist to successfully realizing these projects including insufficiently defined and understood healthcare problems, goals, and workflows, “pilotitis” (ie, the lack of scale-up beyond a pilot phase), and inadequate resources or local interest in sustaining interventions.^5–7^ Integrating principles of health equity into GHI project development and implementation is key to addressing many of these challenges; however, there is little extant literature regarding how an equity-centered approach can reduce barriers to project mobilization and scale up. Our paper builds on existing frameworks on equitably developing and evaluating new technologies in biomedical informatics research^8–10^ and translating such principles into the global health domain. The following paper presents case studies from informatics researchers where such principles were successfully operationalized and provides guidance for how these approaches may be generalized to other projects.

## Setting

A half-day workshop entitled *Advancing health equity in the US and beyond: developing a framework for mutually beneficial informatics collaborations* was held on November 12 at the 2023 AMIA Annual Symposium, the premiere US conference in the medical informatics community that draws over 2300 attendees from academia, industry, non-profits, international and governmental organizations. The workshop was open to all Symposium attendees.

The GHI-WG connected with members of the AMIA community who are active in the health equity and global health informatics domain to identify experts who could share their experiences and facilitate the workshop. Our experts spoke about their experiences working in LMICs and with medically underserved and Indigenous populations in high-income countries. Experts shared knowledge through a collaborative case example from domains that underlie health equity and the GHI lifecycle in academic collaborations. Critical steps in the GHI lifecycle^11^^,^^12^ that are implemented across projects in diverse contexts include:

- Identifying important clinical or public health problems.^13^
- Identifying and/or responding to local health and community leaders, clinicians, and experts.^13^
- Collaboratively identifying strategies to address these problems, including information needs, and suitable health information systems (HIS).^14^
- Collaborating in adapting, improving, implementing, and scaling HIS, and training the full range of users and stakeholders.^15^^,^^16^
- Supporting/assisting in usability testing, evaluating user experience, system performance, and data quality. Supporting evaluation of the impact on health care processes and outcomes and, ideally, impact on the primary health problem.^15^
- Documenting system use, new features, and successes and failures.^15^^,^^17^

## Case studies

Before the workshop, our expert panelists identified and developed case studies for five unique principles that may be incorporated in the GHI lifecycle to develop sustainable, equitable interventions:

1. Inclusion and participation in ethical, sustainable collaborations.
2. Engaging community-based participatory research approaches.
3. Stakeholder engagement.
4. Scalability and sustainability.
5. Representation in knowledge creation.

Each panelist presented a case study for their predefined principle from their own experiences, described methods used to accomplish their initiative’s goals, and highlighted generalizable principles and philosophies embodied in the successes and failures they encountered. Following panel presentations, workshop participants joined break-out discussion groups with one domain per table from each panelist presentation. Panelists and volunteers from the GHI-WG led discussions and actively engaged workshop participants in each group to think critically about the lessons learned from current and past GHI initiatives that participants have experienced. Focus groups then reported out identified principles of collaboration and their application in specific cases to the entire audience of workshop participants.

There were 24 attendees, largely representing academic institutions within the United States, along with members from private industry, the nonprofit sector, and international attendees. Summaries of the panelists’ case studies are presented below. Table 1 presents the problem addressed and the presented solution for each case study and Table S1 presents key points from the discussion groups about challenges faced in these subdomains and how principles of health equity may be incorporated into each stage of the pipeline. Generalizable strategies gleaned from the case studies and key points from discussion groups are discussed below.

**Table 1.** Workshop case study problems and interventions.

**Principle:** Inclusion and Participation in Ethical, Sustainable Collaborations **Moving from paper to digital data in a pediatric clinic in the Dominican Republic (D.R.).** *Presenter: Sansanee Craig, MD*
*Problem addressed:* Informaticists and global health leaders at a U.S.-based academic institution (the Children’s Hospital of Philadelphia- CHOP) partnered with clinical and technical teams in the D.R. to transition from paper to electronic health records to improve outcomes in nutrition, breastfeeding, deworming, anemia, and immunization. *Presented solution:* A point-of-care digital documentation (CommCare) was introduced for home visits and used by the clinic’s senior nurse who does the home visits as well as the clinic’s full-time IT staff. It was shown to be efficient and effective but did not meet the clinic’s sustainability and scalability needs due to the cost of the software and challenges that the staff faced in extracting data. Ongoing collaborations with local technical universities and adherence to global health informatics principles are advancing the development of a sustainable electronic health record system.
**Principle:** Engaging Community-Based Participatory Research Approaches **Journey to working with Indigenous communities.** *Presenter: Oliver J. Bear Don’t Walk IV, PhD (OBDW)*
*Problem addressed:* This case study addresses the challenges in achieving health equity for Indigenous communities, highlighting the misalignment that can occur between academic research priorities and the actual needs of these communities. There is also a noted tension between Western academic standards and the cultural education valued by Indigenous researchers, which can sometimes lead to a loss of cultural identity. Additionally, researchers often face the difficulty of balancing the demands of academia with the need for genuine community engagement that respects and values local expertise. *Presented solution:* One approach to health equity is to work closely with communities to drive research that meets their needs. Centering on Indigenous communities within the US, this case study focused on how GHI researchers can (1) engage humility when working with communities, (2) honor local expertise and traditions, and (3) balance time between academic pressures and meaningfully engaging with communities. The panel presentation by OBDW discussed their journey through academia to work with other Indigenous people, while conveying the importance of humility and reflexivity. While an Indigenous person (Apsáalooke), OBDW emphasized how his time in higher education molded his scientific approach to Western academic standards and noted how his Western education had come at the expense of his cultural education. OBDW ended with reflection and laughter on humbling experiences as they re-integrated into Indigenous spaces.
**Principle:** Stakeholder Engagement **Education and informatics infrastructure in India** *Presenter: Kavishwar B. Wagholikar, MBBS PhD*
*Problem addressed:* The case study highlights the complexities of establishing a sustainable health informatics infrastructure in India through the i2b2 Global South Network. A core challenge lies in creating a framework that not only serves multiple stakeholders, including hospitals, medical schools, industry partners, and government officials, but also addresses the unique needs and concerns of each group. The problem includes balancing value propositions for diverse stakeholders while ensuring data security and privacy, which are critical for trust and long-term collaboration. Furthermore, sustaining these projects beyond initial pilot studies necessitates recognizing and mitigating risks, especially when handling sensitive health data for various applications, from patient care to public health surveillance. *Presented solution:* The solution involves a multi-stakeholder approach that builds infrastructure for health informatics by identifying mutual value propositions and addressing concerns related to data security and privacy. This collaborative framework emphasizes transparency about data collection, usage, and benefits for all parties, ensuring that data-driven applications, such as disease registries or patient-centered apps, clearly communicate their purpose and security measures. By aligning the immediate and long-term benefits with stakeholders’ expectations, including secure data handling and a plan for utilizing insights, the consortium aims to foster a durable and impactful health informatics network. Collaborations with government entities further enhance the infrastructure, enabling efficient disease burden measurement and surveillance, thus addressing broader public health needs in India.
**Principle:** Scalability and Sustainability **EHR Implementation in Rwanda** *Presenter: Hamish Fraser, MBChB MSc FACMI IAHSI*
*Problem addressed:* In Rwanda, the challenge of achieving sustainable local ownership and control over an electronic health records (EHR) system arose early, with a focus on maintaining software and data locally through the OpenMRS EHR platform. Although the Rwandan Ministry of Health successfully employed locally trained developers to implement the system across hundreds of health facilities, issues of reliability and system accessibility persisted. *Presented solution:* Country ownership of the OpenMRS EHR in Rwanda occurred early with Rwanda being one of the 3 founding countries in 2006. Local software and data control was an early priority. This was strengthened by a one-year post-graduate eHealth Software Development Training Program led by Partners in Health (PIH) and the International Development Research Centre (IDRC), that trained 34 EHR developers between 2008 and 2011. The Rwandan Ministry of Health (MOH) employed many of these developers to roll out OpenMRS in more than 350 health facilities, with help from the OpenMRS community, PIH/Inshuti Mu Buzima (PIH/IMB) and the Global Fund. A survey^17^ of 90 OpenMRS users (half clinical, half technical) in 54 health facilities in 2018 showed generally positive views of the system, its benefits and usability. Regular activities included creating or updating records, ordering and viewing lab results and reviewing alerts and reminders. However, 18% of users said the system was available only about half of the time, and 7% said “almost never” or “never”; this is notable as the system had been in use for 6-7 years and was primarily supported by the Rwandan MOH and Rwanda Biomedical Center. While large-scale external funding has helped to expand OpenMRS use in other East African countries, Rwanda has sustained and upgraded the system with less funding, using local leadership and developers along with technical partnership from PIH/IMB and OpenMRS Inc. Local development and leadership is key to local sustainability; however, systems and users can benefit greatly from international collaborations that are, in this case, North-South and South-South (sub-Saharan Africa). Insights from evaluation studies should help improve system design and use and potentially shape future funding. With the rapid growth of interest in AI and machine learning it is critical that countries can understand complete data collection pathways and software as well as access their own data countrywide.
**Principle:** Representation in Knowledge Creation **Scaling data science in response to the COVID-19 pandemic in Sub Saharan Africa** *Presenter: Judy Gichoya and Andrew S. Kanter*
*Problem addressed:* In many publications and presentations on the global status of innovation, Africa is represented as a blank slate, unless the topic is in a more negative light, such as extreme poverty or disease burden. Consequently, it is easy to overlook inclusivity when considering the contribution of global health initiatives in knowledge creation. Nevertheless, numerous important scientific contributions have emerged from Sub-Saharan Africa (SSA) in recent years, notably during the COVID-19 pandemic. For example, the first sequencing of the Omicron variant was performed in SSA,^18^^,^^19^ even though millions of Africans in the continent are yet to receive the SARS-CoV-2 vaccine. Moreover, when news broke on the new variant, the immediate reaction was travel bans to SSA, resulting in economic hardships, an unintended consequence of participating in knowledge creation. *Presented solution:* This presents an important lesson—we must understand and demand that benefits flow to the contributors of knowledge and data in global health informatics, as well as align incentives. It is unlikely that in a future pandemic we will witness the openness and willingness to participate in data sharing and further problem exploration.^20^ Inclusivity in the knowledge creation process means building relationships with low- and middle-income countries and their researchers. While some pillars necessary for this inclusivity, such as open-source and open-science initiatives, are already in place, it is crucial to continue building capacity and fostering intentional inclusivity. This ensures that advancements in technology benefit the patients who contributed the data and prevents global health colonization, to help create a more equitable and representative global health landscape.

The first case study presented an approach for promoting inclusion and participation in the context of an academic collaboration between the Children’s Hospital of Philadelphia (CHOP) and a pediatric clinic in the Dominican Republic to move from paper to digital health data collection. This included allocating substantial time and resources to building relationships with clinic leaders and the Ministry of Health to ensure that the project aligned with current e-Health strategies and that stakeholder input was incorporated into every step of project development and implementation. CHOP researchers also adhered to generalizable best practices listed in the Principles of Digital Development including codesigning with people for inclusion,^21^ and minimized data collection to only what was necessary while respecting data sovereignty and privacy. Focus group participants discussed the need for equitable stakeholder participation to avoid “pilotitis” and practicing radical humility to center local needs and expertise as well as avoid historic paternalism.

The next case study, “Journey to working with indigenous communities” showcased the importance of using cultural humility,^22^ honoring local traditions and expertise, and balancing academic pressures with building meaningful relationships and engaging with communities. Generalizable strategies included emphasizing the need for investigators to independently research the history and customs of the communities they are working with (the presenter did so with Indigenous peoples in the Seattle area), communicating time and resources required for community engagement to the researcher’s academic institution, and building relationships with communities in a way that respects their unique customs and traditions. Participants in this presenter’s focus group discussed community heterogeneity as a challenge in GHI collaborations, the importance of adopting a humble approach when working with community leaders and experts, and utilizing qualitative methods and community-based participatory research to identify community needs that their expertise can address.

The third case study presented an approach to stakeholder engagement in a project to establish a sustainable health informatics infrastructure in India.^23^ Generalizable strategies to identify and engage stakeholders included utilizing a multifaceted approach with one-on-one interactions, focused group meetings, workshops, and conferences. Smaller group interactions are best in initial engagement, while broadening communication to larger activities brings in a greater number and more diverse group of stakeholders. The need for balancing stakeholder needs to facilitate sustainable engagement, including mutual value propositions and concerns about data security and privacy, was emphasized. During the focus group, participants discussed the importance of stakeholder engagement for project management for cooperation and coordination, resolving operational bottlenecks, facilitating scalability, and ensuring sustainability.

The fourth case study presented equity-promoting strategies to ensuring that GHI projects are scalable and sustainable in the context of an OpenMRS EHR implementation project in Rwanda. Researchers prioritized achieving local ownership and control over both the EHR system and data by partnering with local stakeholders and other external organizations that provided resources and training to local developers and implementers. Informatics researchers may apply these processes to their own work by incorporating capacity building into GHI projects to ensure there are resources, educational infrastructure, and local interest in supporting a project beyond its initial ending date. Focus group participants discussed considerations that researchers should take when developing GHI collaborations, including whether their efforts are justifiable, if they enhance care, and if they create benefit for local communities. Participants also emphasized the need for choosing appropriate technologies when working in resource-limited settings.

The final presentation described the lack of representation of researchers from LMICs in scientific literature using a case study of COVID-19 knowledge generation from Sub-Saharan African researchers. This lack of inclusion and representation stimies local expertise, discourages data sharing, and may cause harmful repercussions for countries hosting GHI projects. GHI researchers from high-income countries may promote inclusion in the knowledge generating process from collaborations by utilizing online platforms to disseminate research from their local partners, facilitating networking opportunities for researchers from LMICs, investing in capacity building to enhance collaborators’ scientific writing, and promoting open access journals to mitigate financial barriers to publishing. Discussion group participants identified lessons learned from past GHI projects and existing challenges to increasing representation and inclusiveness in scientific literature. Utilizing open-source software like OMOP and OHDSI, including localized medical terminology in international coding schemes, and avoiding generalizing countries and cultures have fostered inclusiveness. Challenges that remain include limited awareness and engagement with researchers from LMICs in the broader informatics communities, data-sharing difficulties due to varying international laws and policies, and the need to address data colonialism and promote equitable benefits from big datasets for contributing communities.

## Discussion

The AMIA GHI-WG hosted a workshop at the 2023 AMIA Annual Symposium that presented key principles for successful community engagement in health informatics projects with medically underserved populations worldwide using a health equity focus. The workshop aimed to facilitate collaborations, as well as encourage critical thinking about community involvement, needs assessments, and local ownership. Domain experts shared their experiences and approaches in undertaking equitable and mutually beneficial health informatics collaborations with historically marginalized patient populations. Key lessons from the case studies that were presented and the subsequent discussions between participants and experts during the workshop included prioritizing community and stakeholder engagement, centering community voices and needs in project development and implementation, utilizing local technology and expertise, designing for sustainability, and acknowledging and dismantling historical power dynamics in how research is developed and disseminated. Findings from these case studies build upon existing frameworks proposed for equitably conducting biomedical informatics research with a new consideration for working in low-resource settings. Campbell et al. considers the social contexts in which data is collected and the complexities of data bias, highlighting the importance of consideration for the environments in which technology is developed, implemented, and evaluated for GHI projects.^8^ Bear Don’t Walk IV et al. developed a framework that described stages in the ML development process and important ethical principles in the medical domain, in tandem with the baseline principles in inclusiveness and participation described in the case studies that are critical when working in global health informatics research.^9^ Finally, our work builds on Veinot et al.’s study on developing a framework for equitably conducting health informatics research derived from studies conducted in the United States and Canada as it pertains to conducting informatics research in LMICs.^10^ The principles discussed in this workshop are generalizable both to international GHI projects, as well as domestic informatics interventions that work with vulnerable populations and to address health disparities. Researchers and practitioners in the health informatics community can act on this knowledge to identify feasible and appropriate applications of these principles to their own GHI initiatives.

## Supplementary Material

ocaf015_Supplementary_Data

## Data Availability

No formal data was collected during the workshop for analysis. Workshop materials, including the workshop proposal and presentation slides, may be made available on an individual basis upon request to authors.
