# Supplementary material for: Principles and implementation strategies for equitable and representative academic partnerships in global health informatics research
Source: J Am Med Inform Assoc. 2025 Feb 13;32(5):958–63. doi: 10.1093/jamia/ocaf015 (PMC12012363; doi:10.1093/jamia/ocaf015)
Supplement: ocaf015_Supplementary_Data [file ocaf015_supplementary_data.docx]

**Table 1**. Key Lessons and Takeaways from Workshop Focus Groups

| ***Case Study*** | ***Discussion Points and Takeaways*** |
| --- | --- |
| *Baseline Principles for Ethical, Sustainable Collaborations for Inclusion and Participation*:  Moving from paper to digital data in a pediatric clinic in the Dominican Republic | **Avoid mHealth “pilotitis”:**  · Strategically planning for health technology initiatives to scale beyond a pilot stage to sustainable expansion and avoiding siloed data, broken trust, and wasted efforts is necessary.  · Allocating time and effort to forge meaningful relationships with local Ministry of Health individuals is critical to align with existing HICT efforts and national eHealth strategies, as recommended by the WHO for all member countries.  · Co-designing with end users and selecting sustainable and scalable health information technologies is essential.  · Participants noted that minimum necessary data should be collected, with the least possible burden to communities, and with the goal to improve health outcomes.    **Promote mutually beneficial, de-colonized HICT collaborations:**  · Both LMIC and HIC partners can and should benefit equitably from joint implementation initiatives to avoid historic paternalistic dynamics and ensure balanced, just, and impactful outcomes.  · LMIC and HIC partners mutually benefit from unique and diverse perspectives that accelerate high-impact health technology innovation.  · Participants described the importance of practicing radical humility and being intentional about reducing power imbalances in collaborations (i.e. cultivating the ability to say no to a collaboration). |
| *Health Equity*:  Journey to working with indigenous communities | **Leveraging skills and privilege:**  · Researchers should share the role of expert, embrace humility to learn from community leaders and experts, and work closely with communities to drive research to meet their needs.  · Community-based participatory research (CBPR) was discussed as a model for power sharing. Additionally, reflexivity was mentioned as integral for researchers to assess the values they carry when engaging with communities.  **Grounding future research with community knowledge and traditions:**  · Qualitative methods and CBPR-principles can help researchers identify health needs that their expertise can address.  · However, even with these methods and principles in hand, researchers cannot assume that they’re reaching an entire community even if they work with leaders and activists.  **Heterogeneity as a challenge to community-engaged research:**  · Communities cannot be treated as a monolith, regardless of size. For example, within a Tribe, there may be competing views on how knowledge should be shared and categorized in research, all of which are grounded in experience and/or traditions. Even if Tribal leaders support a project, not all members may agree for valid reasons grounded in experience or traditions. By simply working with the group or leaders who support the project, researchers may bias their results, endanger relationships and more importantly harm the community.  · When navigating heterogeneous opinions, concerns, and expertise, researchers will need to update research protocol and results interpretations during the research lifecycle.  · Attendees emphasized the importance of engaging early and often with communities to allow time for these discussions, and to set realistic timelines.    **Challenges to balancing academic pressures:**  · Attendees noted the friction between engaging with communities and the time-sensitive pressures required for promotion and tenure in academia.  · Identifying research questions with communities and then disseminating actionable results takes time and effort beyond the traditional scientific process.  · An open question that remained was how to reward researchers who build respectful, sustainable, and reciprocal relationships within systems that rely on high publication rates and funding end dates. |
| *Stakeholder Engagement*:  Education and informatics infrastructure in India | **Identifying mechanisms for engagement**:  · Includes one-on-one interactions, focused group meetings, workshops, and conferences.  · Smaller group interactions are ideal for implementing activities with initial stakeholders; however, broadening the activity to include other stakeholders with a larger activity like a workshop or conference helps rope in other stakeholders, which may have a more significant value-proposition  · Initial engagements mainly occur through one-on-one virtual meetings and email or phone conversations, and are primarily initiated by word-of-mouth, and these could repeat in tandem with larger events like workshops and conferences to include the other stakeholders.    **Engagement objectives**:  · Resolving operational bottlenecks  · Enhancing cooperation  · Generating credibility for the effort  · Facilitating scalability and ensuring sustainability.  **Project Management**:  ·Engagements with smaller groups is a necessary mechanism to resolve operational issues.  · Project management approaches can enhance cooperation and coordination. Widening the project team to include experts enhances credibility if there is an effort to implement measures to minimize risks.  · A focus to identify and achieve short term and long-term value gains is essential to scale and sustain the project beyond the initial pilots. |
| *Scalability and Sustainability*:  EHR implementation in Rwanda | **Considerations for researchers interested in collaborating on scalable and sustainable GHI partnerships:**  · What is the real value of implementing systems?  · Is that actually going to benefit people?  · How do we do effective evaluation?  · Is this benefitting care? Are our efforts justified?  **Working with resource constraints and local context:**  · It is difficult to get resources in certain settings like Haiti to support systems, so selecting the appropriate system given available resources is essential.  · You can make a system widely available and accessible but need to be able to do updates, etc.  · It is important to remember that in-country systems are the ones who own the data.  · Not having open systems can be problematic.  · Finding the proper balance for these considerations and constraints is an ongoing conversation and area of study. |
| *Representation in Knowledge Creation*:  Scaling data science in response to the COVID-19 pandemic in Sub Saharan Africa | **Lessons learned from past GHI**  · It is critical to disambiguate the broad generalization of countries and cultures in GHI work particularly in Africa.  · Supporting and continuing to expand infrastructure to scale and align representation in knowledge creation is important.  · There has been past success in using open source software (e.g. the OMOP data model, and OHDSI infrastructure to harmonize data from diverse clinical settings and data sources).  · Representation of localized terms in international medical coding schemes is essential for representation and inclusive knowledge generation.  **Challenges**  · Participants noted a lack of awareness and engagement with researchers from under-represented parts of the world in the broader informatics research community.  · Participants also noted how it can be difficult to share data internationally and that countries have diverse laws and policies surrounding data sharing beyond borders.  · It is critical to address the issues of data colonialism and democratizing global health.  · It is essential to ensure that the benefits of big datasets used in global health research flow to the people who contributed the data |
